# Root Morphology of First Permanent Molars Extracted from Adult Dental Patients of Similar Ethnicity in Dar-Es-Salaam, Tanzania

**DOI:** 10.1155/ijod/2531403

**Published:** 2025-02-28

**Authors:** Lorna Celia Carneiro, Risala Shaaban Tekka

**Affiliations:** ^1^Department of Restorative Dentistry, School of Dentistry, Muhimbili University of Health and Allied Sciences, P. O. Box 65014, Dar es Salaam, Tanzania; ^2^Kilwa Road Police Hospital, P. O. Box 9791, Dar es Salaam, Tanzania

## Abstract

**Background:** Root morphology of first permanent molars has been reported to be complex and associated with ethnicity, age, and gender.

**Objective:** To assess the external and internal root morphology of first permanent molars extracted from dental patients of similar ethnicity in Dar es Salaam, Tanzania.

**Methodology:** This descriptive cross-sectional study assessed the external and internal root morphology of first permanent molars extracted from adult patients attending dental clinics in Dar es Salaam. External morphology was visually assessed for root separation or fusion. Separated roots were assessed for the presence and direction of curvature using a graph paper with a drawn vertical and horizontal grid. Computed tomography scanning assessed the internal morphology of teeth embedded in wax blocks. Using transverse slice images at level of pulp floor, number and distance between canal orifices in a root were assessed. Data were analyzed descriptively. For associations, a *p*-value of <0.05% and 95% confidence intervals were used.

**Results:** Of the 384 extracted teeth, 275 (71.6%) fulfilled the inclusion criteria. Majority, (64.2%) of mandibular molars were extracted from patients aged 18–40 years (70.6%) and females (57.6%). Three, (91.2%) and two, (98.0%) separated roots were observed in maxillary and mandibular molars, respectively. Distal curvature of maxillary mesiobuccal root (52.0%) was statistically significantly related to age. Mandibular mesial (83%) roots were curved buccal (*p*  > 0.05). Maxillary mesiobuccal and mandibular mesial roots showed higher proportion of two canal orifices with mean distance of 0.11–0.39 mm between orifices.

**Conclusion:** The majority of maxillary and mandibular first permanent molars from Tanzanian adult dental patients had an external morphology of three and two separated roots with distal curvature of mesiobuccal roots of maxillary molars being statistically significantly related to age. The internal morphology of maxillary mesiobuccal and mandibular mesial roots showed higher proportion of two canal orifices with mean distance varying between 0.11 and 0.39 mm. Variations in root morphology were not related to sex.

## 1. Introduction

Maxillary and mandibular first permanent molars are among the first to erupt in the oral cavity [1] placing them at risk of developing dental caries. For grossly carious teeth, root canal treatment is considered an alternative management to tooth extraction [2]. However, root canal treatment of these teeth poses a problem as their root morphology is known to be complex and variable [3]. Morphological variations reported have been linked to ethnicity [4, 5] differences in age [6–8], and sex [9, 10].

Earlier, root morphology of teeth has been assessed using different methods, such as radiography [11], canal staining, and clearing technique [12–14] and computed tomography scanning [15–17]. Canal staining and clearing technique cause decalcification of the sample, while radiography and conventional computed tomography scanning maintain an intact sample and images of different planes can be viewed with reduced superimposition of surrounding structures [18]. Furthermore, root morphology of teeth has also been assessed using other types of computer tomography like CBCT and Micro-CT [19, 20]. Previous research on external root anatomy of permanent first molars, reported on number of roots [4], root separation [13, 21], or fusion [15, 16, 22] occurrence of additional roots [21, 22], and presence and direction of root curvature [23–25].

Maxillary first permanent molars are known to have three separated roots, mesiobuccal, distobuccal, and palatal roots [13, 21]. However, root fusion has been reported in these teeth with more possibility of different combinations due to the higher number of roots [10]. Presence of curvature of mesiobuccal and distobuccal roots and straight palatal roots have also been reported [5, 17]. Studies on the internal morphology of these teeth report that majority of the mesiobuccal roots contain two canals [17, 26] with mean distance of 0.3ṇ3.8 mm [21, 27] while the distobuccal and palatal roots usually contain a single canal [28, 29].

Mandibular first permanent molars usually have two separated roots; mesial and distal roots [5, 30, 31] with mesial root distally curved and straight distal root [32]. The internal root morphology of this tooth has been reported to have a wide range of variation ranging from two or three canals in the mesial root and one, two, or three canals in the distal root [3, 31, 33] with mean distance of 1–3.5 mm between canals [31, 34].

In Tanzania, there is lack of baseline information with regard to relationship between the root morphology of extracted teeth and age or sex. A previous study that assessed root canal morphology of mandibular molars using the staining and clearing method [35] did not consider age or sex. Furthermore, there is lack of retrievable information on root morphology of permanent maxillary first molars of Tanzanians by age or sex. Therefore, the objective of this study was to provide baseline information on assessed external and internal root morphology of first permanent molars of Tanzanians by age and sex. The obtained findings from this study provide baseline data that enables comparison with published reports from other populations. In addition, knowledge on external and internal morphology of first permanent molars can be used to enhance outcome of root canal treatment in the country.

## 2. Materials and Methods

Following approval from the Ethical Clearance Committee of the Muhimbili University of Health and Allied Sciences (Ref. No. MU/PGS/SAEC/Vol. XVIII), a descriptive cross-sectional study was conducted among adult patients of Tanzanian ethnicity which was verified using patient information. Prior to the study, patients were informed that extracted teeth are normally discarded; however, the collection of these teeth would assist research purposes. Consent was required from those willing to participate, and they were informed that extraction of the tooth was for the relief of pain as treatment alternatives were unavailable.

The United Republic of Tanzania has 31 administrative regions including Dar-es Salaam. The region is mainly commercial and houses people from all other regions and has an estimated population of 5.3 million [36].

**Figure 1:**
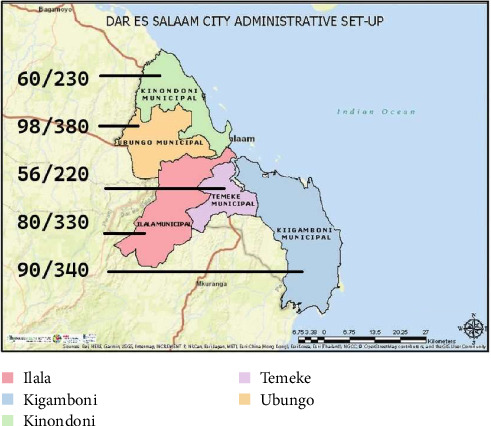
Proportion of participants from each of the five clinics in Dar es Salaam.

The estimated sample size of 384 was obtained using the Cochran formula [37], and an assumed estimated prevalence of 50% of extracted first permanent molars was used in order to get the maximum–minimum sample size. Assuming that there is no difference between assessed variables of external and internal root morphology of first permanent molars of Tanzanians by age (18–77 years) or sex (males = 126; females = 149), the sample size estimation was not stratified to these two individual-level variables. Proportionate sampling of study participants was based on number of attendees at each clinic over a period of 2 months. Proportion of participants based on the estimated sample size from each of the five clinics in Dar es Salaam is shown in Figure 1.

Included in the study, were adult patients of Tanzanian descent aged at least 18 years who consented and whose extracted first permanent molars had intact roots and pulp chamber. Excluded from the study were teeth that could compromise findings due to destroyed external or internal morphology, namely teeth that were root canal treated, had fractured root/s or destroyed floor of pulp chamber.

**Figure 2:**
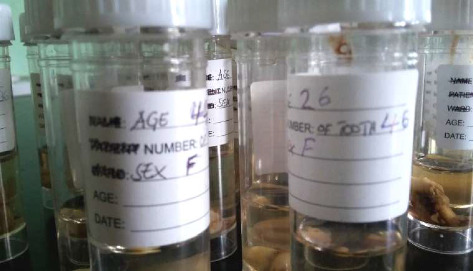
Containers used to collect samples with label indicating patient's age, sex, and notation of extracted tooth.

The principal investigator trained one research assistant at each clinic on method of obtaining of consent and collection of samples. Each extracted tooth was placed into a plastic container (ATICO Medical Pvt. Ltd. Item Code: AM-806) half-filled with 10% formalin (Central Pathology Laboratory of Muhimbili National Hospital) and having a blank label for indicating patients age in years, sex (male or female) and extracted tooth number (Figure 2).

Processing of samples was done at the preclinical dental laboratory of the School of Dentistry, Muhimbili University of Health and Allied Sciences (MUHAS). Using disposable gloves (Neogloves, Lot 6547, Sri Trang Gloves Company Limited, Thailand), formalin from each container was discarded into a sink and tooth and container were rinsed using tap water. They were then half filled with 5% sodium hypochlorite solution (NCG Chemical Industries Ltd, Dar es Salaam, Tanzania) to allow for disinfection and dissolving of adherent soft tissues. After 3 h of standing, the hypochlorite solution was discarded and tap water was used to rinse contents. Adherent soft tissues, bone, or calculus were removed by root planning prior to sorting according to inclusion criteria.

Using information on the label, age was recorded and further grouped into 18–40 and 41–77 years based on the assumption that the canal system is completely defined by 40 years [8].

Sex was recorded as male or female and using the two-digit notation tooth type was recorded (16, 26, 36 or 46) and regrouped into maxillary (16 and 26) or mandibular (36 and 46) molars.

**Figure 3:**
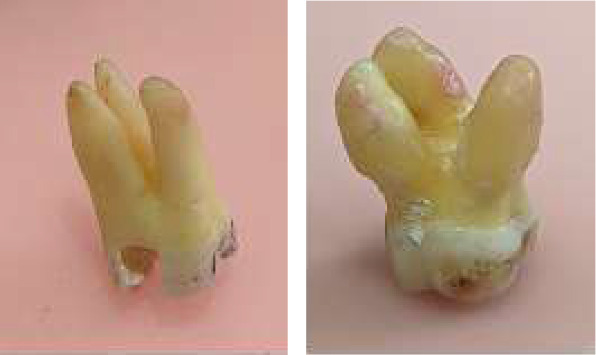
Extracted maxillary first molar with (a) separated roots and (b) fused roots.

External root morphology of each tooth was visually assessed to determine if roots were separated (clear demarcation) or fused (dehiscence to one another). Separated roots were counted and recorded using Arabic numerals, while fused roots were counted as one root (Figure 3).

**Figure 4:**
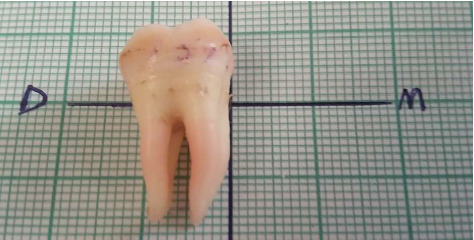
Alignment of each root and direction of curvature following placement on graph paper with vertical and horizontal grid indicating distal (D) and mesial (M) orientation.

A graph paper of 10 mm x10 mm squares (Jamana Printers, Dar es Salaam) with a drawn horizontal and vertical grid at the center was used to determine the alignment (straight or curved) and direction of curvature of each separated root. The buccal surface of each tooth was placed on the graph paper and long axis of tooth was oriented to the vertical grid while the cemento-enamel junction was aligned on the horizontal grid with correct mesial (M) and distal (D) surface orientation [23] as shown in Figure 4. A root was recorded to be straight when the entire root was aligned to the vertical axis and curved when it deviated from the vertical axis. Direction of curvature was determined with regard to tooth surfaces.

**Figure 5:**
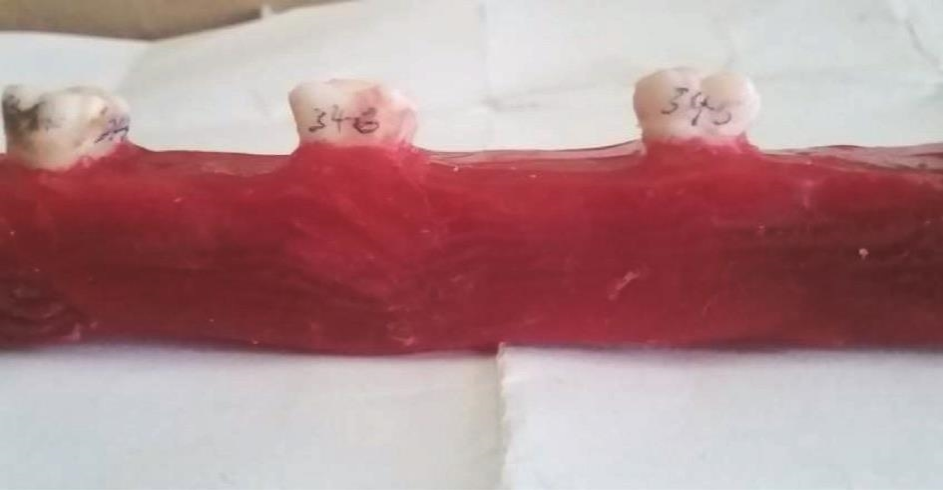
A total of 10 extracted first permanent molars of similar tooth type were numbered and embedded in prepared utility wax blocks of 30 cm x3 cm x3 cm.

Prior to the assessment of internal root morphology, utility wax sheets (KERR brand, Romulus, Mich. 48174) were used to prepare blocks measuring 30 cm x3 cm x3 cm. Ten extracted teeth of similar tooth type were sequentially numbered and embedded into the blocks up to the cemento-enamel junction maintaining a distance of 2 cm and with similar buccal/palatal and mesial/distal orientation (Figure 5).

**Figure 6:**
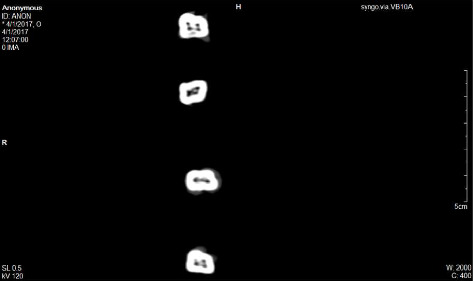
An image of a CT slice at level of floor of pulp chamber showing number of root canal orifices in each root of a tooth.

The prepared blocks were arranged on the table of the computed tomography (CT) machine (Siemen Somatom definition Flash, 128 slice) located at the Radiology Department, Muhimbili National Hospital (MNH) [38]. Scanning was performed using a distance of 5 cm, slice thickness of 0.5 mm and exposure time of 8 s, 120 kV and 35 mA. When a full slice was completed, the image was stored and the motorized bed was moved forward incrementally into the gantry until the desired number of slices were obtained. Using the image processing device software, brightness, and contrast of the three-dimensional images were adjusted to enable optimal visualization and slice reconstruction at a 1 : 1 ratio. The slice images of teeth were orientated to enable viewing from the top to bottom and a transverse plane at level of floor of the pulp chamber was used to assess number of root canal orifices in a root (Figure 6).

**Figure 7:**
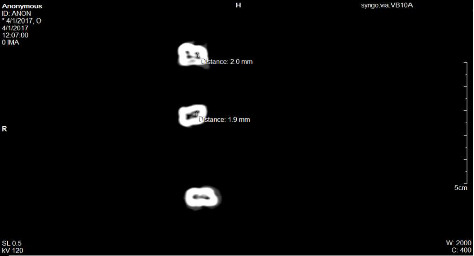
A CT slice at the level of the floor of the pulp chamber of teeth showing number of canal orifices and the distance between the canal orifices in a root of a tooth.

Using a transverse plane at level of floor of the pulp chamber the distance between orifices in each root was measured in millimeters (mm) by use of the software (Figure 7).

Pretesting of all tools was done using extracted teeth that were not included in the study. Intra-examiner's reliability was assessed by a repeat evaluation of every tenth tooth and the respective Cohen's Kappa scores of 0.93, 0.95, 0.89, and 0.87 were obtained for number of roots, root curvature, number of root canal orifices and the mean distances, respectively, between orifices per root.

The main variables that were assessed included patients age (18–40 and 41–77 years), sex (male and female), and extracted tooth type (maxillary −16 and 26; mandibular −36 and 46). Morphology of separated or fused roots, number of roots per tooth, presence/absence of root curvature, direction of root curvature, and number of root canal orifices in each root by age and sex was assessed using cross tabulations. The univariable analysis included frequencies especially when describing the morphology of first permanent molars of study participants and the median (with interquartile range) when summarizing the skewed quantitative variables. Furthermore, research team used the Pearson Chi-Square test when assessing the association between categorical variables. Distance between root canal orifices in a root was determined using the means and associated standard deviations. The level of significance throughout the analysis was set at 5%, and all the analyses were done using the SPSS software for Windows version 20.0.

## 3. Results

Of the 384 first permanent molars extracted from Tanzanians, 275 fulfilled the inclusion criteria giving a response rate of 71.6%. However, based on the preestimated prevalence of extracted first molars of 50% and the observed prevalence from the sample data of 25.6%, we get the effect size of 48.8%. Therefore, the power of the study was at least 99.9%.

The median age of patients was 26.0 (interquartile range = 13.0) years and ranged between 18 and 77 years. The majority, (*n* = 223; 84.7%) of the extracted first permanent molars were from patients aged 18–40 years, females (*n* = 149; 54.2%). and from the mandible (*n* = 162; 58.9%) (Table 1).

Shown in Table 2 is the distribution of location of extracted first permanent molars by age and sex. A higher proportion of the extracted first permanent mandibular molars were from those aged 41–77 years 26 (61.9%) and among females 90 (60.4%).

Shown in Table 3 is the distribution of number of roots of extracted first permanent molars by age and sex. There were more maxillary molars with three separated roots (*n* = 103; 91.2%) than those with two roots (*n* = 10; 8.8%). Proportion of two separated roots (*n* = 159; 98.1%) in mandibular molars was more than those with one (*n* = 2; 1.3%) and three (*n* = 1; 0.6%) roots.

Majority of the maxillary molars in the younger age group of 18–40 years had three roots (*n* = 87; 89.7%) and two roots (*n* = 10; 10.3%) unlike the older age group who only had three roots. There was more or less an equal distribution of maxillary molars with three roots among males and females; however, females having two roots were more than males. Many more extracted mandibular molars from patients aged 18–40 years had two separated roots (*n* = 159; 98.1%) with few having one (*n* = 2; 1.5%) and three roots (*n* = 1; 0.7%) while all of the older age group had two roots. Unlike females who had one (*n* = 2; 2.2%), two (*n* = 87; 96.7%) and three (*n* = 1; 1.1%) roots, all of the males had two roots in mandibular molars.

Shown in Table 4 is the distribution of alignment of separated roots of extracted first permanent molars. Mesiobuccal roots of maxillary 48 (46.6%) and mesial roots of mandibular 149 (93.7%) molars had the highest proportion of curved roots.

Distribution of alignment of separated roots of extracted first permanent molars by age and sex is shown in Table 5. Maxillary teeth of the younger age group had a statistically significantly higher proportion of curved mesiobuccal roots (*n* = 46; 95.8%) in comparison to their counterparts (p < 0.05). Curved mesial roots of mandibular molars were more in the younger (*n* = 125; 83.9%) than older age group. Roots of maxillary teeth showed no association between sex of patient and their curvature.

**Figure 8:**
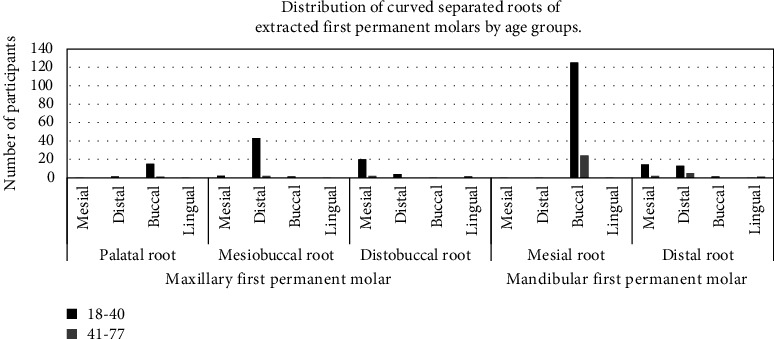
Distribution of direction of curved separated roots of extracted first permanent molars by age groups.

The distribution of direction of curved separated roots of extracted first permanent molars by age is shown in Figure 8. Compared to the older age group a higher proportion of the younger age group had buccal, distal, and mesial curvature of the palatal, mesiobuccal, and distobuccal roots of maxillary molars, respectively. Also, more of the younger than older age group had buccal curvature of the mesial root of mandibular molars and both mesial and distal curvature of the distal roots in comparison to the older age group.

**Figure 9:**
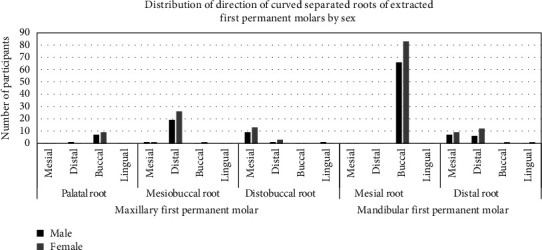
Distribution of direction of curved separated roots of extracted first permanent molars by sex.

Shown in Figure 9 is the distribution of direction of curved separated roots of extracted first permanent molars by sex. More females than males had buccal, distal, and mesial curvature of palatal, mesiobuccal, and distobuccal root of maxillary molars and buccally curved mesial roots of mandibular molars.

**Figure 10:**
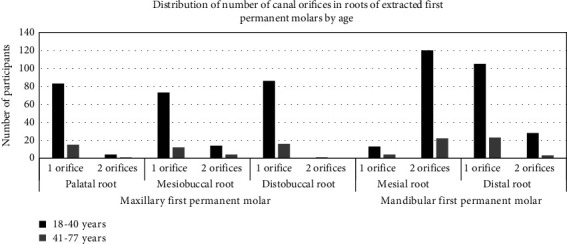
Distribution of number of canal orifices in roots of extracted first permanent molars by age.

Figure 10 shows the distribution of number of canal orifices in roots of extracted first permanent molars by age. Many more of the younger age group had one orifice in the palatal, mesiobuccal and distobuccal roots of maxillary molars and two orifices in mesiobuccal and palatal roots in comparison to the older age group. In mandibular molars age group of 1840 years had higher number of two orifices in mesial root and one orifice in the distal root when compared to the older age group.

**Figure 11:**
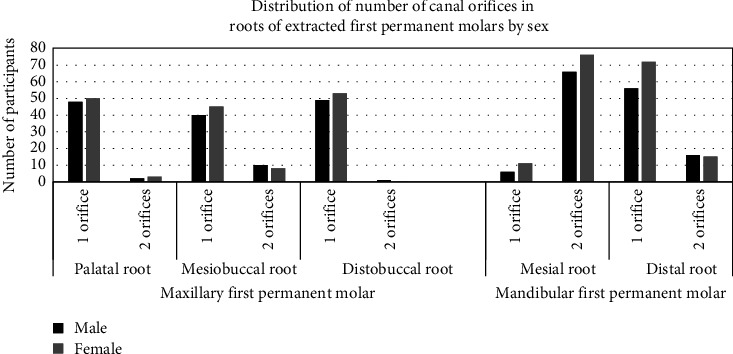
Distribution of number of canal orifices in roots of extracted first permanent molars by sex.

Distribution of number of canal orifices in roots of extracted first permanent molars by sex is shown in Figure 11. Higher proportion of females than males had one orifice in palatal, mesiobuccal, and distobuccal roots of maxillary molars. In mandibular molars, proportion of females with two orifices in mesial root and one orifice in distal root was higher than males. Males had a higher proportion of two orifices in mesiobuccal root of maxillary molars and distal root of mandibular molars in comparison to females.

Table 6 shows the mean distance between canal orifices in each root of extracted first permanent molars. In extracted first maxillary molars, the mean distance between canal orifices in palatal, mesiobuccal, and distobuccal roots was 0.22 ± 0.15, 0.18 ± 0.06 and 0.11 mm, respectively. The mean distance between canal orifices of mesial and distal roots of mandibular first permanent molars was of 0.39 ± 1.26 and 0.23 ± 0.08.

## 4. Discussion

This hospital-based cross-sectional study assessed variations in external and internal root morphology of first permanent molars extracted from adult patients of similar ethnicity in Dar es Salaam, Tanzania. Henceforth, practitioners should consider the highlighted anatomical variations in root morphology when providing root canal treatment.

Generalization of findings from this study should be interpreted with caution as employed study design and sampling procedure could have introduced bias. In addition, being a hospital-based study, it involved only patients attending dental clinics in each municipality of Dar es Salaam and Tanzania as a whole. Furthermore, power analysis determined that the reduced sample size was adequate to detect meaningful differences at 99.9%.In accordance with a previous hospital-based cross-sectional study on dental patients in Tanzania [39], this study also found a higher number of patients in the younger age group of 18–40 years. This could be a reflection of the general population which is composed mainly of the younger adults [36]. These findings are a reflection that tooth extraction among younger adults needs to be minimized by preventive strategies coupled with root canal treatment.

More than half of the participants in this study were females unlike other studies that reported a male predominance [40, 41]. It is known that oral habits and practices of females place them at a higher risk of developing dental caries [39] and probably why they utilize dental services much more than any other group [42] suggestive that preventive measures should be enhanced to this group.

In agreement with another study [43], this study also found that nearly two-thirds of the extracted first permanent molars were mandibular molars. First permanent mandibular molars are known to erupt earlier than their maxillary counterparts, and it is possible that their longer exposure in the oral cavity predispose them to risk of developing dental caries [4, 34] which has been reported to be the main reason for tooth extraction [42]. Root canal treatment of these teeth should be considered as an alternative treatment.

Contrasting to studies in Burmese [44] and Thai [13] populations that reported presence of three separate roots in maxillary first molars, a study among Brazilians reported an even lower prevalence of three-rooted maxillary first molars and they attributed these findings to racial differences [45]. Similar to findings of this study, maxillary molars having two and three roots was also reported in other ethnic populations, namely, Ugandan [12], Iranian [46], Saudi Arabian [47], Greek [17], Korean [48] and Chinese population [49]. Variation in number of roots between studies could be related to the assessment method [48]. Furthermore, this study could have unintentionally excluded teeth with aberrations.

Nearly all of the mandibular molars in this sampled Tanzanian population had two roots. Presence of two roots was also reported among 97%, 74%, 70%, and 65.56% of populations of Sri Lanka [30], Brazil [50], China [51], and Iran [52], respectively. However, the 0.6% of mandibular teeth with three roots in this study was much lower than the 29% reported among Iranians [52]. Differences in the reported number of roots in different populations could be a true finding and should be considered when performing root canal treatment. Furthermore, the assessment methods used in the evaluation of root morphology in the different studies may have varying precision.

Similar to this study, fused roots of maxillary and mandibular first molars were also reported in studies done in Uganda [12] and Iran [53]. Contrastingly, studies in Burmese [44] and Thai [13] populations reported no fusion of roots. The method used to classify fused roots could be the reason for differences in studies. Some studies report root fusion when there is complete radicular fusion [54] unlike in this study that reported fusion when roots showed dehiscence to one another. Root canal treatment of fused teeth is often challenging due to canal shape abnormalities [55].

The statistically significant higher proportion of curved mesiobuccal roots (95.8%) of maxillary first molars among the younger age group is in agreement with studies that reported variations by age [789]. Contrastingly, a study among the Turkish population reported no occurrence of root curvature in roots of teeth in either jaw [56]. A higher occurrence of curved roots in maxilla than mandible was reported among Croation adults [25]. Distal curvature of mesiobuccal roots of permanent maxillary first molars was also reported among Kenyans [57], while buccal curvature of mesial roots of mandibular first molars was reported among Chinese [58]. Various methods for determining root canal curvature are described in the literature; however, there is no consensus on which method to use [25, 59]. Regardless, practitioners should consider the possibility of curved roots and direction of curvature when performing root canal treatment. It is possible that root curvature among the younger age group and females in this study is a true finding despite the lack of statistical difference and could be related to differences in biting forces [60]. However, the presence of root curvature by age and sex can be researched further.

Variation in number of canal orifices in maxillary teeth in this study was also reported in studies done in Iran [61], Saudi Arabia [47], and Jordan [31]. Presence of two canal orifice in the mesiobuccal root of maxillary teeth has also been reported by other researchers [13, 62], and the higher occurrence [11] may been attributed to the assessment method [63]. The variation in the number of root canal orifices in the mesiobuccal root of maxillary first permanent molars could be a true finding among Tanzanians and should be considered when performing root canal treatment.

In accordance to findings of studies among the Sri Lankan population [30], Chinese population [54], and Brazilian population [64], this study also found that majority of the mesial roots of first mandibular molars had two canal orifices. Contrastingly, the occurrence of four canals, two mesial and two distal canals, was reported in the Jordanian population [31] and Saudi Arabian Population [65]. Differences in studies could be related to method utilized in detectability of canal orifices [66], and clinicians should be encouraged to routinely assess a tooth for additional canals prior to execution of root canal treatment.

In comparison to the reported mean horizontal inter-orifice distance of 1.21 ± 0.5 mm between the MB1 and MB2 orifices of maxillary first molars [27], this study reported an even lesser inter-orifice distance of 0.09–0.37 mm. Contrastingly, an even larger inter-orifice distance of 2.55 ± 0.57 mm was reported in an Iranian population [21]. Comparative studies on mean distance for mesial and distal roots of mandibular first molars were scarce; however, variations in mean distance between root canal orifices could be related to the computer software used. In addition, prior to root canal treatment, practitioners should estimate the inter-orifice distance by assessing pulp chamber size, pulpal obliteration, and root canal configuration [67].

Contrary to studies that report differences in root morphology of first permanent molars by sex [9, 10] this study on ethnic Tanzanian patients of African origin did not show statistical significant differences by sex. Regardless practitioners should consider the possibility of differences of root curvature of mesiobuccal maxillary root and mesial mandibular root between different sexes. Furthermore, the presence of a second canal is a reported variation between males and females that renders due consideration when performing root canal treatment. However, for the success of root canal treatment reported variations should be considered.

## 5. Conclusion

The majority of maxillary and mandibular first permanent molars from Tanzanian adult dental patients had an external morphology of three and two separated roots with distal curvature of mesiobuccal roots of maxillary molars being statistically significantly related to age. The internal morphology of maxillary mesiobuccal and mandibular mesial roots showed higher proportion of two canal orifices with mean distance varying between 0.11 and 0.39 mm. Variations in root morphology were not related to sex.

## 6. Recommendations

• However, for the success of root canal treatment reported variations should be considered.
• Whenever possible, clinicians performing root canal treatment are encouraged to use three-dimensional diagnostic techniques in the identification of external and internal root canal morphology.
• Further research on external and internal root morphology of permanent first molars could affirm the lack of influence of age and sex reported in this study.

## Figures and Tables

**Table 1:** Distribution of study sample by assessed variables (*N* = 275).

Variable	Number	Percent (95% CI)
Age group (years)		
18–40	233	84.7 (79.9, 88.8)
41–77	42	15.3 (11.2, 20.1)
Sex		
Male	126	45.8 (39.8, 51.9)
Female	149	54.2 (48.1, 60.2)
Location of first permanent molar		
Maxilla	113	41.1 (35.2, 47.2)
Mandible	162	58.9 (52.8, 64.8)

**Table 2:** Distribution of location of extracted first permanent molars by age and sex (*N* = 275).

Variable	Extracted first permanent molars	
	Maxilla (*n* = 113)	Mandible (*n* = 162)
	Number (%); (95% CI)	Number (%); (95% CI)
Age group (years)		
18–40	97 (41.6); (35.2, 48.3)	136 (58.4); (51.8, 64.8)
41–77	16 (38.1); (23.6, 54.4)	26 (61.9); (45.6, 76.4)
Sex		
Male	54 (42.9); (34.1, 51.9)	72 (57.1); (48.1, 65.9)
Female	59 (39.6); (31.7, 47.9)	90 (60.4); (52.1, 68.3)

**Table 3:** Distribution of number of roots of extracted first permanent molars by age and sex (*N* = 275).

Variables	Number of roots in extracted teeth					
	Maxillary molars			Mandibular molars		
	Two (*n* = 10; 8.8%)	Three (*n* = 103; 91.2%)	One 2 (1.3)		Two 159 (98.1)	Three 1 (0.6)
	Number (%); (95% CI)	Number (%); (95% CI)	Number (%); (95% CI)		Number (%); (95% CI)	Number (%); (95% CI)
Age (years)						
18–40	10 (10.3); (5.1, 18.1)	87 (89.7); (81.9, 94.9)	2 (1.5); (0.2, 5.2)		133 (97.8); (93.7, 99.6)	1 (0.7); (0.1, 4.1)
41–77	0 (0)	16 (100)	0 (0)		26 (100)	0 (0)
Sex						
Male	4 (7.4); (2.1, 17.8)	50 (92.6); (82.1, 97.9)	0 (0)		72 (100)	0 (0)
Female	6 (10.2); (3.8, 2.1)	53 (89.8); (79.2, 96.2)	2 (2.2); (0.3, 7.8)		87 (96.7); (90.6, 99.3)	1 (1.1); (0.03, 6.1)

**Table 4:** Distribution of alignment of separated roots of extracted first permanent molars (*N* = 262).

First permanent molars with separated roots	Alignment	
	Straight	Curved
	Number (%); (95% CI)	Number (%); (95% CI)
Maxillary (*n* = 103)		
Palatal root	86 (83.5); (74.9, 90.1)	17 (16.5); (9.9, 25,1)
Mesiobuccal	55 (53.4); (43.3, 63.3)	48 (46.6); (36.7, 56.7)
Distobuccal	76 (73.8); (64.2, 81.9)	27 (26.2); (18.1, 35.8)
Mandibular (*n* = 159)		
Mesial	10 (6.3); (3.1,11.3)	149 (93.7); (88.7, 96.9)
Distal	123 (77.4); (70.1,83.6)	36 (22.6); (16.4, 29.9)

**Table 5:** Distribution of alignment of separated roots of extracted first permanent molars by age and sex (*n* = 262).

Variables	Age groups (years)		Sex	
	18–40	41–77	Male	Female
	Number (%); (95% CI)	Number (%); (95% CI)	Number (%); (95% CI)	Number (%); (95% CI)
Maxillary teeth				
*Palatal root*				
Straight	71 (82.6); (72.8, 89.9)	15 (17.4); (10.1, 27.1)	42 (48.9); (37.9, 59.7)	44 (51.1); (40.1, 62.1)
Curved	16 (94.1); (71.3, 99.8)	1 (5.9); (0.2, 28.7)	8 (47.1); (22.9, 72.2)	9 (52.9); (27.8, 77.1)
*Mesiobuccal root*				
Straight	41 (74.5); (61, 85.3)	14 (25.5); (14.7, 39.0)	30 (54.6); (40.6, 68.0)	25 (45.4); (31.9, 59.4)
Curved	46 (95.8)*⁣*^*∗*^; (85.8, 99.5)	2 (4.2); (0.5, 14.3)	20 (41.7); (27.6, 56.8)	28 (58.3); (43.2, 72.4)
*Distobuccal root*				
Straight	62 (81.6); (71.0, 89.6)	14 (18.4); (10.5, 28.9)	39 (51.3); (39.6, 62.9)	37 (48.7); (37.0, 60.4)
Curved	25 (92.6); (75.7, 99.1)	2 (7.4); (0.9, 24.3)	11 (40.7); (22.4, 61.2)	16 (59.3); (38.8, 77.6)
Mandibular teeth				
*Mesial root*				
Straight	8 (80.0); (44.4, 97.5)	2 (20.0); (2.5, 55.6)	6 (60.0); (26.2, 87.8)	4 (40.0); (12.2, 73.8)
Curved	125 (83.9); (77, 89.4)	24 (16.1); (10.6, 23.0)	65(43.6); (35.5, 51.9)	84 (56.4); (48.0, 64.5)
Distal root				
Straight	105 (85.4); (77.7, 91.1)	18 (14.6); (8.9, 22.1)	58 (47.2); (38.1, 56.4)	65 (52.8); (43.6, 61.9)
Curved	28 (77.8); (60.8, 89.9)	8 (22.2); (10.1, 39.1)	13 (36.1); (20.8, 53.8)	23 (36.9); (46.2, 79.2)

*⁣* ^*∗*^ *p* ≤ 0.05.

**Table 6:** Distribution of mean orifice distance in millimeters (mm) between roots of first permanent molars by age and sex.

First permanent molars with more than one canal in separated roots	Lowest and highest value (mm)	Mean distance (SD)
Maxillary molars		
Palatal (*n* = 5)		
MP—DP	0.12–0.5	0.23 (0.16)
Mesiobuccal (*n* = 18)		
MB1–MB2	0.09–0.37	0.18 (0.07)
Distobuccal (*n* = 1)		
DB1DB2	0.11	0.11 (0.0)
Mandibular molars		
Mesial (*n* = 142)		
MB–ML	0.10–0.55	0.39 (1.27)
Distal (*n* = 31)		
DB–DL	0.10–0.36	0.24 (0.07)

Abbreviation: SD, standard deviation

## Data Availability

The authors confirm that the data supporting the findings of this study are available within the article and upon reasonable request raw data will be made available by the corresponding author.

## References

[1] Yamunadevi A., Pratibha R., Rajmohan M. (2021). First Molars in Permanent Dentition and Their Malformations in Various Pathologies: A Review. *Journal of Pharmacy and Bioallied Sciences*.

[2] Barbhai S., Shetty R., Joshi P. (2022). Evaluation of Root Anatomy and Canal Configuration of Human Permanent Maxillary First Molar Using Cone-Beam Computed Tomography: A Systematic Review. *International Journal of Environmental Research and Public Health*.

[3] Weng X.-L., Yu S.-B., Zhao S.-L. (2009). Root Canal Morphology of Permanent Maxillary Teeth in the Han Nationality in Chinese Guanzhong Area: A New Modified Root Canal Staining Technique. *Journal of Endodontics*.

[4] Alrahabi M., Zafar M. (2015). Evaluation of Root Canal Morphology of Maxillary Molars Using Cone Beam Computed Tomography. *Pakistan Journal of Medical Sciences*.

[5] Nur B., Ok E., Altunsoy M. (2019). Evaluation of the Root and Canal Morphology of Mandibular Permanent Molars in a South-Eastern Turkish Population Using Cone-Beam Computed Tomography. *European Journal of Dentistry*.

[6] Reis A., Grazziotin-Soares R., Barletta F., Fontanella V., Mahl C. (2013). Second Canal in Mesiobuccal Root of Maxillary Molars Is Correlated With Root Third and Patient Age: A Cone-Beam Computed Tomographic Study. *Journal of Endodontics*.

[7] Wu D., Zhang G., Liang R. (2017). Root and Canal Morphology of Maxillary Second Molars by Cone-Beam Computed Tomography in a Native Chinese Population. *Journal of International Medical Research*.

[8] Peiris H., Pitakotuwage T., Takahashi M., Sasaki K., Kanazawa E. (2008). Root Canal Morphology of Mandibular Permanent Molars at Different Ages. *International Endodontic Journal*.

[9] Sert S., Bayirli G. (2004). Evaluation of the Root Canal Configurations of the Mandibular and Maxillary Permanent Teeth by Gender in the Turkish Population. *Journal of Endodontics*.

[10] Martins J., Marques D., Francisco H., Caramês J. (2018). Gender Influence on the Number of Roots and Root Canal System Configuration in Human Permanent Teeth of a Portuguese Subpopulation. *Quintessence International*.

[11] Dosumu O. O., Abiodun-Solanke I. M. F., Shaba P. O., Ajayi D. M. (2008). Prevalence of Additional Canals in Maxillary First Molars in a Nigerian Population. *The Journal of Contemporary Dental Practice*.

[12] Rwenyonyi C., Kutesa A., Muwazi L., Buwembo W. (2009). Root and Canal Morphology of Mandibular First and Second Permanent Molar Teeth in a Ugandan Population. *Odontology*.

[13] Alavi A. M., Opasanon A., Ng Y. L., Gulabivala K. (2002). Root and Canal Morphology of Thai Maxillary Molars. *International Endodontic Journal*.

[14] Al-Qudah A., Awawdeh L. (2009). Root and Canal Morphology of Mandibular First and Second Molar Teeth in a Jordanian Population. *International Endodontic Journal*.

[15] Cleghorn B., Christie W., Dong C. (2006). Root and Root Canal Morphology of the Human Permanent Maxillary First Molar: A Literature Review. *Journal of Endodontics*.

[16] Estrela C., Rabelo L., de Souza J. (2015). Frequency of Root Canal Isthmi in Human Permanent Teeth Determined by Cone-Beam Computed Tomography. *Journal of Endodontics*.

[17] Nikoloudaki G., Kontogiannis T., Kerezoudis N. (2015). Evaluation of the Root and Canal Morphology of Maxillary Permanent Molars and the Incidence of the Second Mesiobuccal Root Canal in Greek Population Using Cone-Beam Computed Tomography. *The Open Dentistry Journal*.

[18] Nalçaci R., Öztürk F., Sökücü O. (2010). A Comparison of Two-Dimensional Radiography and Threedimensional Computed Tomography in Angular Cephalometric Measurements. *Dentomaxillofacial Radiology*.

[19] Reda R., Di Nardo D., Zanza A. (2024). Upper First and Second Molar Pulp Chamber Endodontic Anatomy Evaluation According to a Recent Classification: A Cone Beam Computed Tomography Study. *Journal of Imaging*.

[20] Reda R., Zanza A., Bhandi S. (2022). Surgical-Anatomical Evaluation of Mandibular Premolars by CBCT Among the Italian Population. *Dental and Medical Problems*.

[21] Faramarzi F., Vossoghi M., Shokri A. (2015). Cone Beam Computed Tomography Study of Root and Canal Morphology of Maxillary First Molar in an Iranian Population. *Avicenna Journal of Dental Research*.

[22] Pécora J., Woelfel J., Sousa Neto M. (1991). Morphologic Study of the Maxillary Molars. 1. External Anatomy. *Brazilian Dental Journal*.

[23] Nyaga J. M. (2010). External and Internal Root Morphology of First Permanent Molars in a Kenyan Population.

[24] Hamasha A., Al-Khateeb T., Darwazeh A. (2002). Prevalence of Dilaceration in Jordanian Adults. *International Endodontic Journal*.

[25] Malcić A., Jukić S., Brzović V. (2006). Prevalence of Root Dilaceration in Adult Dental Patients in Croatia. *Oral Surgery, Oral Medicine, Oral Pathology, Oral Radiology, and Endodontology*.

[26] Wang Y.-L., Chang H.-H., Kuo C.-I. (2013). A Study on the Root Canal Morphology of Primary Molars by High-Resolution Computed Tomography. *Journal of Dental Sciences*.

[27] Spagnuolo G., Ametrano G., D’Antò V. (2012). Microcomputed Tomography Analysis of Mesiobuccal Orifices and Major Apical Foramen in First Maxillary Molars. *The Open Dentistry Journal*.

[28] Degerness R., Bowles W. (2010). Dimension, Anatomy and Morphology of the Mesiobuccal Root Canal System in Maxillary Molars. *Journal of Endodontics*.

[29] Briseño-Marroquín B., Paqué F., Maier K., Willershausen B., Wolf T. (2015). Root Canal Morphology and Configuration of 179 Maxillary First Molars by Means of Micro-Computed Tomography: An Ex Vivo Study. *Journal of Endodontics*.

[30] Peiris R., Takahashi M., Sasaki K., Kanazawa E. (2007). Root and Canal Morphology of Permanent Mandibular Molars in a Sri Lankan Population. *Odontology*.

[31] Al-Khriesat A., Al-Ghnanaeem M., Al Saddi R. (2014). The Incidence of a Fourth Canal in Maxillary and Mandibular First Molars in a Group of Jordanians: A Clinical Study. *Journal of the Royal Medical Services*.

[32] Djalma J., Rivera S., Neto M., Costa L. (1996). External and Internal Anatomy of Mandibular Molars. *Brazilian Dental Journal*.

[33] Gu Y., Lu Q., Wang H. (2010). Root Canal Morphology of Permanent Three-Rooted Mandibular First Molars--Part I: Pulp Floor and Root Canal System. *Journal of Endodontics*.

[34] Zhang X., Xiong S., Ma Y. (2015). A Cone-Beam Computed Tomographic Study on Mandibular First Molars in a Chinese Subpopulation. *PLoS ONE*.

[35] Madjapa H., Minja I. (2018). Root Canal Morphology of Native Tanzanian Permanent Mandibular Molar Teeth. *Pan African Medical Journal*.

[36] Ministry of Finance and Planning, Tanzania National Bureau of Statistics, Office of the Chief Government Statistician Zanzibar (2022). The 2022 Population and Housing Census: Age and Sex Distribution Report Tanzania Mainland.

[37] Cochran W. G. (1977). *Sampling Techniques*.

[38] Barakzai S., Barnett T. (2015). Computed Tomography and Scintigraphy for Evaluation of Dental Disease in the Horse. *Equine Veterinary Education*.

[39] Sachedina T., Sohal K. S., Owibingire S. S., Hamza O. J. M. (2023). Reasons for Delay in Seeking Treatment for Dental Caries in Tanzania. *International Dental Journal*.

[40] Nyorobi J. M., Carneiro L. C., Kabulwa M. N. (2018). Knowledge and Practices on Periodontal Health among Adults, Misungwi, Tanzania. *International Journal of Dentistry*.

[41] Nimako-Boateng J., Owusu-Antwi M., Nortey P. (2016). Factors Affecting Dental Diseases Presenting at the University of Ghana Hospital. *SpringerPlus*.

[42] Kikwilu E. N., Frencken J. E., Mulder J., Masalu J. R. (2009). Barriers to Restorative Care as Perceived by Dental Patients Attending Government Hospitals in Tanzania. *Community Dentistry and Oral Epidemiology*.

[43] Almugla Y. (2021). Prevalence of Missing First Permanent Molars in a Selected Population in a University Dental Clinic Setting: A Retrospective Radiographic Study. *International Journal of Clinical Pediatric Dentistry*.

[44] Ng Y.-L., Aung T. H., Alavi A., Gulabivala K. (2001). Root and Canal Morphology of Burmese Maxillary Molars. *International Endodontic Journal*.

[45] Silva E., Nejaim Y., Silva A. (2014). Evaluation of Root Canal Configuration of Maxillary Molars in a Brazilian Population Using Cone-Beam Computed Tomographic Imaging: An in Vivo Study. *Journal of Endodontics*.

[46] Rouhani A., Bagherpour A., Akbari M. (2014). Cone-Beam Computed Tomography Evaluation of Maxillary First and Second Molars in Iranian Population: A Morphological Study. *Iranian Endodontic Journal*.

[47] Mashyakhy M., Awawdeh M., Abu-Melha A. (2022). Anatomical Evaluation of Root and Root Canal Configuration of Permanent Maxillary Dentition in the Population of the Kingdom of Saudi Arabia. *BioMed Research International*.

[48] Kim Y., Lee S., Woo J. (2012). Morphology of Maxillary First and Second Molars Analyzed by Cone-Beam Computed Tomography in a Korean Population: Variations in the Number of Roots and Canals and the Incidence of Fusion. *Journal of Endodontics*.

[49] Zheng Q., Wang Y., Zhou X. (2010). A Cone-Beam Computed Tomography Study of Maxillary First Permanent Molar Root and Canal Morphology in a Chinese Population. *Journal of Endodontics*.

[50] Silva E., Nejaim Y., Silva A., Haiter-Neto F., Cohenca N. (2013). Evaluation of Root Canal Configuration of Mandibular Molars in a Brazilian Population by Using Cone-Beam Computed Tomography: An in Vivo Study. *Journal of Endodontics*.

[51] Zhang R., Wang H., Tian Y.-Y., Yu X., Hu T., Dummer P. M. H. (2011). Use of Cone-Beam Computed Tomography to Evaluate Root and Canal Morphology of Mandibular Molars in Chinese Individuals. *International Endodontic Journal*.

[52] Shahi S., Yavari H., Rahimi S., Torkamani R. (2008). Root Canal Morphology of Human Mandibular First Permanent Molars in an Iranian Population. *Journal of Dental Research Dental Clinics Dental Prospects*.

[53] Ghoncheh Z., Zade B., Kharazifard M. (2017). Root Morphology of the Maxillary First and Second Molars in an Iranian Population Using Cone Beam Computed Tomography. *Journal of Dentistry*.

[54] Zhang Q., Chen H., Fan B., Fan W., Gutmann J. L. (2014). Root and Root Canal Morphology in Maxillary Second Molar With Fused Root from a Native Chinese Population. *Journal of Endodontics*.

[55] Rudagi K., Rudagi B. M., Metgud S., Wagle R. (2012). Endodontic Management of Mandibular Second Molar Fused to a Supernumerary Tooth, Using Spiral Computed Tomography as a Diagnostic Aid: A Case Report. *Case Reports in Dentistry*.

[56] Miloglu O., Cakici F., Caglayan F., Yilmaz A. B., Demirkaya F. (2009). The Prevalence of Root Dilacerations in a Turkish Population. *Medicina Oral Patología Oral y Cirugia Bucal*.

[57] Nyaga J., Maina S., Gathece L., Okoth J. (2016). Root Curvature in the Maxillary First Permanent Molars in a Kenyan Population. *African Journal of Oral Health Science*.

[58] Fu Y., Gao Y., Gao Y. (2022). Three-Dimensional Analysis of Coronal Root Canal Morphology of 136 Permanent Mandibular First Molars by Micro-Computed Tomography. *Journal of Dental Sciences*.

[59] Chohayeb A. (1983). Dilaceration of Permanent Upper Lateral Incisors: Frequency, Direction, and Endodontic Treatment Implications. *Oral Surgery, Oral Medicine, Oral Pathology*.

[60] Palinkas M., Nassar M., Cecílio F. (2010). Age and Gender Influence on Maximal Bite Force and Masticatory Muscles Thickness. *Archives of Oral Biology*.

[61] Khademi A., Zamani Naser A., Bahreinian Z. (2017). Root Morphology and Canal Configuration of First and Second Maxillary Molars in a Selected Iranian Population: A Cone-Beam Computed Tomography Evaluation. *Iranian Endodontic Journal*.

[62] Ilich M. K. A (2014). *External and Internal Root Morphology of Second Permanent Molars of a Kenyan Population*.

[63] Alaçam T., Tinaz A., Genç O., Kayaoglu G. (2008). Second Mesiobuccal Canal Detection in Maxillary First Molars Using Microscopy and Ultrasonics. *Australian Endodontic Journal*.

[64] Caputo B., Noro Filho G., de Andrade Salgado D. M. (2016). Evaluation of the Root Canal Morphology of Molars by Using Cone-Beam Computed Tomography in a Brazilian Population: Part I. *Journal of Endodontics*.

[65] Mack F., Mundt T., Budtz-Jørgensen E. (2003). Prosthodontic Status among Old Adults in Pomerania, Related to Income, Education Level, and General Health (Results of the Study of Health in Pomerania, SHIP). *The Journal of Prosthetic Dentistry*.

[66] Keleş A., Keskin C. (2017). Detectability of Middle Mesial Root Canal Orifces by Troughing Technique in Mandibular Molars: A Micro-Computed Tomo Graphic Study. *Journal of Endodontics*.

[67] Gupta S., Saxena P., Jain S., Jain D. (2011). Prevalence and Distribution of Selected Developmental Dental Anomalies in an Indian Population. *Journal of Oral Science*.

